# Investigation of *Cytotoxic T-lymphocyte antigen-4* polymorphisms in non-small cell lung cancer: a case-control study

**DOI:** 10.18632/oncotarget.20638

**Published:** 2017-09-04

**Authors:** Shuchen Chen, Yafeng Wang, Yu Chen, Jihong Lin, Chao Liu, Mingqiang Kang, Weifeng Tang

**Affiliations:** ^1^ Department of Thoracic Surgery, Fujian Medical University Union Hospital, Fuzhou, Fujian Province, China; ^2^ Department of Cardiology, The People's Hospital of Xishuangbanna Dai Autonomous Prefecture, Jinghong, Yunnan Province, China; ^3^ Department of Medical Oncology, Fujian Cancer Hospital, Fujian Medical University Cancer Hospital, Fuzhou, Fujian Province, China; ^4^ Department of Cardiothoracic Surgery, Affiliated People's Hospital of Jiangsu University, Zhenjiang, Jiangsu Province, China; ^5^ Key Laboratory of Ministry of Education for Gastrointestinal Cancer, Fujian Medical University, Fuzhou, Fujian Province, China; ^6^ Fujian Key Laboratory of Tumor Microbiology, Fujian Medical University, Fuzhou, Fujian Province, China

**Keywords:** CTLA-4, polymorphism, non-small cell lung cancer, immune

## Abstract

The objective of this case-control study was to extensively explore the relationship of *Cytotoxic T-lymphocyte antigen-4* (CTLA-4) tagging polymorphisms with susceptibility to non-small-cell lung cancer (NSCLC). We recruited 521 sporadic NSCLC cases and 1,030 non-cancer controls. The genotypes of CTLA-4 rs16840252 C>T, rs231775 G>A, rs3087243 G>A and rs733618 T>C polymorphisms were evaluated using a custom-by-design 48-Plex SNPscan Kit. Our findings revealed there was no statistically significant difference in CTLA-4 genotypes distribution among NSCLC patients and non-cancer controls. Similar findings were observed in the logistic regression analyses. However, the stratified analyses suggested CTLA-4 rs733618 vatiants were correlated with the development of NSCLC in ≥ 60 years subgroup (TC vs. TT: adjusted OR = 1.45, 95% CI = 1.04–2.02, *P* = 0.030) and even drinking subgroup (TC vs. TT: adjusted OR = 2.27, 95% CI = 1.11–4.60, *P* = 0.024 and TC/CC vs. TT: adjusted OR = 2.26, 95% CI = 1.15–4.43, *P* = 0.018). In conclusion, the present case-control study highlights that the CTLA-4 rs733618 T>C polymorphism was associated with the development of NSCLC in ≥ 60 years and even drinking subgroups. A fine-mapping study with functional assessment is necessary to confirm or refute our findings.

## INTRODUCTION

In 2012, an estimated 1,824,700 new lung cancer (LC) cases occurred worldwide, accounting for approximately 13% of overall cancer diagnosis [1]. LC was the most common malignancy and the first leading cause of cancer-related death among males in 2012; in addition, it was the second cancer-related death among females [1]. Thus, exploration of potential heredity factors that might affect the risk of LC, especially non-small-cell LC (NSCLC), which was the most common subtype of cases, attracted our interest. We focused on the costimulatory molecules of immunoglobulin superfamily which regulate T-cell activation and proliferation.

Cytotoxic T-lymphocyte antigen-4 (CTLA-4), a member of the immunoglobulin superfamily, is also known as CD152. In generally, CTLA-4 is expressed on activated T cells and negatively regulate the proliferation and the activation of T cells [2–4]. CTLA-4 competes with CD28 and binds to B7.1 and B7.2 which are costimulatory molecules expressed on antigen-presenting cells. In addition, the affinity between CTLA-4 and B7 molecules is higher than that of CD28 with B7 molecules [5, 6]. The interaction of CTLA-4–B7 leads to the repression of T cells at the G_1_ phase and the down-regulated expression of interleukin-2 (IL-2) and IL-2 receptor [7]. This interaction can also induce activated T cells to FAS-independent apoptosis, and then further restrain T lymphocytes.

CTLA-4, a immunoregulatory molecule, is encoded by a gene on chromosome 2q33. A number of single-nucleotide polymorphisms (SNPs) in *CTLA-4* gene have been established. Song et al. reported that *CTLA-4* +49A>G polymorphism was a prognostic predictor for advanced NSCLC [8]. In addition, Antczak et al. found that CTLA-4 expression was significantly correlated with CTLA-4 TT genotype (-318C/T). Recently, several case-control studies focused on the relationship of *CTLA-4* SNPs with the risk of NSCLC [8–12]. However, due to the limited sample size and the number of study, the association between *CTLA-4* SNPs and NSCLC susceptibility was not well understood. The objective of this case-control study was to extensively explore the relationship of *CTLA-4* rs16840252 C>T, rs231775 G>A, rs3087243 G>A and rs733618 T>C polymorphisms with susceptibility to NSCLC.

## RESULTS

### Demographic characteristics

This case-control study comprised 521 NSCLC cases and 1,030 control subjects. The NSCLC patients comprised 287 males and 234 females, while the non-cancer control subjects were 588 males and 442 females. The mean age and SD in the NSCLC patient was 59.76 ± 10.71 years and that was 60.34 ± 9.11 years in controls. Gender and age were well-matched between the groups (*P* = 0.453 and *P* = 0.843, respectively, Table 1). All 521 confirmed cases of NSCLC were sporadic. The genotyping successful rates were shown in Table 2 and they ranged from 99.81% to 99.94%. The values of MAF in control subjects were very similar to the data for Chinese (Table 2). The genotype frequencies of *CTLA-4* rs16840252 C>T, rs231775 G>A, rs3087243 G>A and rs733618 T>C polymorphisms in controls reached Hardy-Weinberg equilibrium (HWE).

**Table 1:** Distribution of selected demographic variables and risk factors in NSCLC cases and controls

Variable	Overall Cases (*n* = 521)	Overall Controls (*n* = 1,030)	*P*^a^
	*n* (%)	*n* (%)	
Age (years)	59.76 ±10.71	60.34 ± 9.11	0.268
Age (years)			0.843
< 60	238 (45.68)	476 (46.21)	
≥ 60	283 (54.32)	554 (53.79)	
Sex			0.453
Male	287 (55.09)	588 (57.09)	
Female	234 (44.91)	442 (42.91)	
Smoking status			**< 0.001**
Never	317 (60.84)	828 (80.39)	
Ever	204 (39.16)	202 (19.61)	
Alcohol use			**< 0.001**
Never	444 (85.22)	949 (92.14)	
Ever	77 (14.78)	81 (7.86)	
BMI (kg/m^2^)	23.00 (±3.03)	23.84 (±3.06)	**< 0.001**
BMI (kg/m^2^)			
< 24	337 (64.68)	547 (53.11)	**< 0.001**
≥ 24	184 (35.32)	483 (46.89)	
Lymph node status			
Positive	200 (38.39)		
Negative	314 (60.27)		
Unknown	7 (1.34)		
TMN stage			
I + II	315 (60.46)		
III + IV	206 (39.54)		
Type of NSCLC			
Adenocarcinoma	415 (79.65)		
Squamous cell carcinoma	85 (16.31)		
Others	21 (4.03)		

^a^ Two-sided *χ*^2^ test and student *t* test;

Bold values are statistically significant (*P* < 0.05);

BMI, body mass index.

NSCLC: non-small-cell lung cancer

**Table 2:** Primary information for *CTLA-4* polymorphisms (rs3087243 G>A, rs16840252 C>T, rs733618 T>C and rs231775 G>A)

Genotyped SNPs	*CTLA-4*rs3087243 G>A	*CTLA-4*rs16840252 C>T	*CTLA-4*rs733618 T>C	*CTLA-4*rs231775 G>A
Chromosome	2	2	2	2
Function	nearGene-3	nearGene-5	nearGene-5	missense
Chr Pos (NCBI Build 38)	203874196	203866796	203866221	203867991
MAF^a^ for Chinese in database	0.183	0.122	0.390	0.314
MAF in our controls (*n* ***=***1,040)	0.182	0.121	0.407	0.300
*P* value for HWE^b^ test in our controls	0.532	0.146	0.314	0.950
Genotyping method	SNPscan	SNPscan	SNPscan	SNPscan
% Genotyping value	99.94%	99.94%	99.94%	99.81%

^a^MAF: minor allele frequency;

^b^HWE: Hardy–Weinberg equilibrium.

### Association of *CTLA-4* rs16840252 C>T, rs231775 G>A, rs3087243 G>A and rs733618 T>C Polymorphisms with NSCLC

Table 3 demonstrated the detailed frequencies of *CTLA-4* rs16840252 C>T, rs231775 G>A, rs3087243 G>A and rs733618 T>C genotypes. Results of the single locus analyses were summarized in Table 4. We found no statistically significant difference in *CTLA-4* genotype distribution among NSCLC patients and non-cancer controls. The similar findings were observed in the logistic regression analyses.

**Table 3:** The frequencies of *CTLA-4* rs3087243 G>A, rs16840252 C>T, rs733618 T>C and rs231775 G>A polymorphisms in different NSCLC subgroups

Genotype	NSCLC Cases (*n* = 521)		Adenocarcinoma (*n* = 415)		Non-adenocarcinoma (*n* = 106)		Controls (*n* = 1,030)	
	*n*	%	*n*	%	*n*	%	*n*	%
rs3087243 G>A								
GG	344	66.03	273	65.78	71	66.98	686	66.67
GA	160	30.71	131	31.57	29	27.36	312	30.32
AA	17	3.26	11	2.65	6	5.66	31	3.01
A allele	194	18.62	153	18.43	41	19.34	374	18.17
rs16840252 C>T								
CC	400	76.78	323	77.83	77	72.64	791	76.87
CT	111	21.31	86	20.72	25	23.58	228	22.16
TT	10	1.92	6	1.45	4	3.77	10	0.97
T allele	131	12.57	98	11.81	33	15.57	248	12.05
rs733618 T>C								
TT	172	33.01	138	33.25	34	32.08	370	35.96
TC	267	51.25	214	51.57	53	50.00	481	46.74
CC	82	15.74	63	15.18	19	17.92	178	17.30
C allele	431	41.36	340	40.96	91	42.92	837	40.67
rs231775 G>A								
GG	254	48.85	206	49.76	48	45.28	504	49.03
GA	219	42.12	175	42.27	44	41.51	431	41.93
AA	47	9.04	33	7.97	14	13.21	93	9.05
A allele	313	30.10	241	29.11	72	33.96	617	30.01

**Table 4:** Logistic regression analyses of association between *CTLA-4* polymorphisms and risk of NSCLC

Genotype	Overall NSCLC cases (*n* = 521) vs. controls (*n* = 1030)				Adenocarcinoma (*n* = 415) vs. controls (n=1030)				Non-adenocarcinoma (*n* = 106) vs. controls (*n* = 1030)			
	Crude OR (95% CI)	P	Adjusted OR^a^ (95% CI)	P	Crude OR (95% CI)	P	Adjusted ORa (95% CI)	P	Crude OR (95% CI)	P	Adjusted OR^a^ (95% CI)	P
rs3087243 G>A												
Additive model	1.02 (0.81–1.29)	0.839	1.02 (0.81–1.30)	0.852	1.06 (0.83–1.35)	0.663	1.07 (0.83–1.37)	0.627	0.90 (0.57–1.41)	0.646	0.79 (0.48–1.29)	0.342
Homozygote model	1.10 (0.60–2.01)	0.769	1.05 (0.56–1.97)	0.872	0.89 (0.44–1.80)	0.752	0.89 (0.44–1.83)	0.759	1.87 (0.76–4.64)	0.175	1.51 (0.55–4.16)	0.429
Dominant model	1.03 (0.82–1.29)	0.801	1.03 (0.81–1.29)	0.836	1.04 (0.82–1.32)	0.747	1.05 (0.82–1.34)	0.708	0.99 (0.65–1.51)	0.948	0.86 (0.54–1.37)	0.517
Recessive model	1.09 (0.60–1.98)	0.788	1.05 (0.56–1.95)	0.890	0.88 (0.44–1.76)	0.711	0.88 (0.43–1.78)	0.714	1.93 (0.79–4.74)	0.151	1.63 (0.60–4.43)	0.343
rs16840252 C>T												
Additive model	0.96 (0.75–1.25)	0.779	0.96 (0.74–1.26)	0.777	0.93 (0.70–1.22)	0.584	0.91 (0.68–1.21)	0.525	1.13 (0.70–1.81)	0.619	1.24 (0.73–2.09)	0.426
Homozygote model	1.98 (0.82–4.80)	0.130	1.37 (0.55–3.44)	0.499	1.47 (0.53–4.08)	0.458	1.12 (0.39–3.18)	0.838	4.12 (1.26–13.44)	0.019	2.46 (0.62–9.80)	0.200
Dominant model	1.01 (0.78–1.29)	0.967	0.98 (0.76–1.28)	0.896	0.95 (0.72–1.24)	0.694	0.92 (0.70–1.22)	0.563	1.25 (0.80–1.97)	0.329	1.32 (0.806–2.18)	0.279
Recessive model	1.99 (0.83–4.82)	0.126	1.38 (0.55–3.46)	0.487	1.50 (0.54–4.14)	0.439	1.14 (0.40–3.24)	0.808	4.00 (1.23–12.98)	0.021	2.34 (0.59–9.23)	0.225
T allele												
rs733618 T>C												
Additive model	1.20 (0.95–1.51)	0.133	1.21 (0.95–1.55)	0.122	1.20 (0.93–1.54)	0.166	1.20 (0.93–1.56)	0.167	1.20 (0.77–1.89)	0.424	1.30 (0.79–2.12)	0.305
Homozygote model	0.99 (0.72–1.37)	0.969	1.07 (0.77–1.49)	0.687	0.95 (0.67–1.35)	0.779	1.01 (0.71–1.44)	0.959	1.17 (0.65–2.10)	0.612	1.50 (0.78–2.88)	0.228
Dominant model	1.14 (0.91–1.42)	0.251	1.17 (0.93–1.48)	0.175	1.13 (0.89–1.43)	0.330	1.15 (0.90–1.47)	0.270	1.19 (0.78–1.82)	0.427	1.34 (0.84–2.14)	0.220
Recessive model	0.89 (0.67–1.19)	0.438	0.96 (0.71–1.29)	0.762	0.86 (0.63–1.17)	0.329	0.91 (0.66–1.25)	0.546	1.04 (0.62–1.76)	0.871	1.28 (0.72–2.29)	0.400
rs231775 G>A												
Additive model	1.01 (0.81–1.26)	0.942	0.99 (0.78–1.24)	0.901	0.99 (0.78–1.26)	0.951	0.98 (0.76–1.25)	0.839	1.08 (0.70–1.65)	0.737	1.04 (0.65–1.67)	0.861
Homozygote model	1.00 (0.69–1.47)	0.989	0.93 (0.63–1.39)	0.735	0.87 (0.57–1.33)	0.516	0.83 (0.54–1.29)	0.417	1.59 (0.84–3.00)	0.154	1.23 (0.61–2.50)	0.567
Dominant model	1.01 (0.82–1.24)	0.946	0.98 (0.78–1.22)	0.837	0.97 (0.77–1.22)	0.802	0.95 (0.75–1.20)	0.677	1.16 (0.78–1.74)	0.463	1.08 (0.69–1.67)	0.737
Recessive model	1.00 (0.69–1.44)	0.996	0.94 (0.64–1.38)	0.753	0.87 (0.58–1.32)	0.513	0.84 (0.55–1.29)	0.435	1.53 (0.84–2.79)	0.166	1.20 (0.62–2.36)	0.588

^a^ Adjusted for age, sex, smoking status, alcohol use and BMI status

In a stratified analysis by cancer type of NSCLC, logistic regression analyses indicated that there was no difference in genotype distribution of *CTLA-4* rs231775 G>A, rs16840252 C>T, rs3087243 G>A and rs733618 T>C polymorphisms among different NSCLC types and controls (Table 4).

### Association of *CTLA-4* rs16840252 C>T, rs231775 G>A, rs3087243 G>A and rs733618 T>C Polymorphisms with NSCLC in a stratification analysis

Tables 5–7 summarized the genotype frequencies of *CTLA-4* rs3087243 G>A, rs16840252 C>T and rs231775 G>A polymorphisms in the stratified analyses by gender, age, BMI, drinking and smoking status. We found no difference in genotype distribution of *CTLA-4* rs16840252C>T, rs231775 G>A and rs3087243 G>A polymorphisms among NSCLC cases and the control subjects in any subgroup.

**Table 5:** Stratified analyses between CTLA-4 rs3087243 G>A polymorphism and NSCLC risk by sex, age, BMI, smoking status and alcohol consumption

Variable	*CTLA-4* rs3087243 G>A (case/control)^a^			Adjusted OR^b^ (95% CI); P			
	GG	GA	AA	additive model	homozygote model	Dominant model	Recessive model
Sex							
Male	184/387	94/178	9/22	1.16 (0.84–1.61); *P*: 0.381	0.76 (0.33–1.75); *P*: 0.513	1.11 (0.81–1.52); *P*: 0.531	0.72 (0.31–1.66); *P*: 0.442
Female	160/299	66/134	8/9	0.89 (0.62–1.28); *P*: 0.541	1.66 (0.62–4.46); *P*: 0.312	0.94 (0.67–1.33); *P*: 0.734	1.72 (0.65–4.59); *P*: 0.277
Age							
< 60	162/320	72/142	4/13	1.09 (0.76–1.55); *P*: 0.653	0.67 (0.21–2.20); *P*: 0.514	1.05 (0.74–1.49); *P*: 0.785	0.66 (0.20–2.13); *P*: 0.483
≥ 60	182/366	88/170	13/18	1.00 (0.72–1.39); *P*: 0.987	1.33 (0.62–2.86); *P*: 0.459	1.04 (0.76–1.42); *P*: 0.825	1.33 (0.63–2.84); *P*: 0.456
Smoking status							
Never	207/556	100/249	10/22	1.06 (0.79–1.41); *P*: 0.708	1.21 (0.55–2.65); *P*: 0.632	1.07 (0.81–1.42); *P*: 0.647	1.19 (0.55–2.58); *P*: 0.665
Ever	137/130	60/63	7/9	0.90 (0.58–1.39); *P*: 0.630	0.77 (0.27–2.14); *P*: 0.610	0.88 (0.58–1.34); *P*: 0.556	0.79 (0.29–2.19); *P*: 0.653
Alcohol consumption							
Never	292/636	138/285	14/27	1.01 (0.78–1.31); *P*: 0.927	1.04 (0.52–2.06); *P*: 0.916	1.01 (0.79–1.30); *P*: 0.918	1.03 (0.52–2.04); *P*: 0.926
Ever	52/50	22/27	3/4	0.96 (0.46–1.99); *P*: 0.911	0.76 (0.15–3.91); *P*: 0.747	0.93 (0.46–1.87); *P*: 0.842	0.77 (0.15–3.90); *P*: 0.756
BMI (kg/m^2^)							
< 24	223/350	104/178	10/18	0.91 (0.67–1.24); *P*: 0.546	0.95 (0.42–2.18); *P*: 0.911	0.91 (0.68–1.23); *P*: 0.546	0.98 (0.43–2.23); *P*: 0.967
≥ 24	121/336	56/134	7/13	1.21 (0.82–1.78); *P*: 0.343	1.13 (0.42–3.06); *P*: 0.806	1.20 (0.83–1.74); *P*: 0.342	1.07 (0.40–2.86); *P*: 0.985

^a^ The genotyping was successful in 521 (100.00%) NSCLC cases, and 1029 (99.90%) controls for *CTLA-4* rs3087243 G>A; ^b^ Adjusted for age, sex, BMI, smoking status and alcohol consumption (besides stratified factors accordingly) in a logistic regression model.

**Table 6:** Stratified analyses between *CTLA-4* rs16840252 C>T polymorphism and NSCLC risk by sex, age, BMI, smoking status and alcohol consumption

Variable	*CTLA-4* rs16840252 C>T (case/control)^a^			Adjusted OR^b^ (95% CI); *P*			
	CC	CT	TT	Additive model	Homozygote model	Dominant model	Recessive model
Sex							
Male	214/462	65/121	8/4	1.23 (0.85–1.78); *P*: 0.269	3.22 (0.90–11.54); *P*: 0.073	1.31 (0.92–1.87); *P*: 0.136	3.08 (0.86–10.99); *P*: 0.084
Female	186/329	46/107	2/6	0.70 (0.47–1.04); *P*: 0.074	0.39 (0.07–2.04); *P*: 0.262	0.68 (0.46–1.00); *P*: 0.052	0.43 (0.08–2.24); *P*: 0.315
Age							
< 60	177/367	56/102	5/6	1.08 (0.73–1.60); *P*: 0.701	1.27 (0.37–4.37); *P*: 0.702	1.09 (0.75–1.60); *P*: 0.652	1.25 (0.37–4.28); *P*: 0.722
≥ 60	223/424	55/126	5/4	0.84 (0.58–1.22); *P*: 0.366	1.49 (0.38–5.92); *P*: 0.570	0.87 (0.61–1.25); *P*: 0.448	1.55 (0.39–6.13); *P*: 0.534
Smoking status							
Never	245/631	70/188	2/8	0.94 (0.68–1.29); *P*: 0.704	0.46 (0.10–2.22); *P*: 0.333	0.91 (0.67–1.25); *P*: 0.579	0.47 (0.10–2.25); *P*: 0.340
Ever	155/160	41/40	8/2	1.04 (0.64–1.71); *P*: 0.874	4.10 (0.84–19.97); *P*: 0.081	1.18 (0.74–1.90); *P*: 0.485	4.07 (0.84–19.72); *P*: 0.082
Alcohol consumption							
Never	343/731	91/210	10/7	0.96 (0.72–1.27); *P*: 0.759	2.04 (0.74–5.62); *P*: 0.167	1.00 (0.76–1.32); *P*: 0.996	2.06 (0.75–5.66); *P*: 0.162
Ever	57/60	20/18	0/3	1.05 (0.50–2.23); *P*: 0.890	-	0.90 (0.44–1.87); *P*: 0.785	-
BMI(kg/m^2^)							
< 24	256/410	74/127	7/9	0.98 (0.70–1.38); *P*: 0.901	1.02 (0.37–2.84); *P*: 0.974	0.98 (0.71–1.36); *P*: 0.906	1.02 (0.37–2.84); *P*: 0.967
≥ 24	144/381	37/101	3/1	0.94 (0.61–1.45); *P*: 0.773	5.70 (0.53–61.80); *P*: 0.152	0.99 (0.65–1.53); *P*: 0.977	5.77 (0.53–62.46); *P*: 0.149

^a^The genotyping was successful in 521 (100.00%) NSCLC cases, and 1029 (99.90%) controls for *CTLA-4* rs16840252 C>T; ^b^Adjusted for age, sex, BMI, smoking status and alcohol consumption (besides stratified factors accordingly) in a logistic regression model.

**Table 7:** Stratified analyses between *CTLA-4* rs231775 G>A polymorphism and NSCLC risk by sex, age, smoking status and alcohol consumption

Variable	*CTLA-4* rs231775 G>A (case/control)^a^			Adjusted OR^b^ (95% CI); *P*			
	GG	GA	AA	Additive model	Homozygote model	Dominant model	Recessive model
Sex							
Male	132/292	123/241	31/54	1.16 (0.85–1.60); *P*: 0.355	1.20 (0.71–2.02); *P*: 0.500	1.18 (0.87–1.60); *P*: 0.286	1.12 (0.68–1.85); *P*: 0.655
Female	122/212	96/190	16/39	0.81 (0.58–1.14); *P*: 0.233	0.64 (0.34–1.20); *P*: 0.164	0.78 (0.56–1.08); *P*: 0.129	0.70 (0.38–1.29); *P*: 0.254
Age							
< 60	114/237	103/195	21/43	1.13 (0.80–1.59); *P*: 0.485	1.01 (0.56–1.82); *P*: 0.980	1.11 (0.80–1.53); *P*: 0.550	0.95 (0.54–1.68); *P*: 0.863
≥ 60	140/267	116/236	26/50	0.89 (0.65–1.23); *P*: 0.487	0.88 (0.51–1.51); *P*: 0.640	0.90 (0.66–1.21); *P*: 0.469	0.93 (0.55–1.57); *P*: 0.781
Smoking status							
Never	155/407	137/346	24/73	0.98 (0.74–1.30); *P*: 0.893	0.82 (0.50–1.37); *P*: 0.454	0.96 (0.73–1.25); *P*: 0.734	0.83 (0.51–1.36); *P*: 0.461
Ever	99/97	82/85	23/20	0.96 (0.63–1.45); *P*: 0.838	1.10 (0.57–2.15); *P*: 0.773	0.99 (0.67–1.46); *P*: 0.942	1.13 (0.59–2.13); *P*: 0.717
Alcohol consumption							
Never	218/470	183/397	42/80	0.95 (0.74–1.22); *P*: 0.696	1.04 (0.68–1.60); *P*: 0.842	0.97 (0.77–1.23); *P*: 0.791	1.07 (0.71–1.61); *P*: 0.751
Ever	36/34	36/34	5/13	1.13 (0.56–2.29); *P*: 0.726	0.38 (0.12–1.20); *P*: 0.099	0.90 (0.47–1.74); *P*: 0.756	0.36 (0.12–1.08); *P*: 0.068
BMI(kg/m^2^)							
< 24	162/249	148/238	27/58	0.99 (0.74–1.34); *P*: 0.966	0.69 (0.41–1.16); *P*: 0.160	0.93 (0.70–1.23); *P*: 0.590	0.69 (0.42–1.13); *P*: 0.144
≥ 24	92/255	71/193	20/35	0.94 (0.64–1.36); *P*: 0.732	1.50 (0.81–2.80); *P*: 0.200	1.04 (0.73–1.47); *P*: 0.848	1.56 (0.86–2.85); *P*: 0.146

^a^The genotyping was successful in 520 (99.81%) NSCLC cases, and 1028 (99.81%) controls for *CTLA-4* rs231775 G>A; ^b^Adjusted for age, sex, BMI, smoking status and alcohol consumption (besides stratified factors accordingly) in a logistic regression model;

As shown in Table 8, the stratified analyses suggested *CTLA-4* rs733618 vatiants were correlated with the development of NSCLC in ≥ 60 years subgroup (TC vs. TT: adjusted odds ratio (OR) = 1.45, 95% confidence intervals (CI) = 1.04–2.02, *P* = 0.030) and even drinking subgroup (TC vs. TT: adjusted OR = 2.27, 95% CI = 1.11–4.60, *P* = 0.024 and TC/CC vs. TT: adjusted OR = 2.26, 95% CI = 1.15–4.43, *P* = 0.018).

**Table 8:** Stratified analyses between *CTLA-4* rs733618 T>C polymorphism and NSCLC risk by sex, age, smoking status and alcohol consumption

Variable	*CTLA-4* rs733618 T>C (case/control)^a^			Adjusted OR^b^ (95% CI); *P*			
	TT	TC	CC	Additive model	Homozygote model	Dominant model	Recessive model
Sex							
Male	94/211	149/271	44/105	1.28 (0.91–1.79); *P*: 0.156	1.06 (0.67–1.67); *P*: 0.810	1.22 (0.88–1.67); *P*: 0.234	0.91 (0.61–1.38); *P*: 0.669
Female	78/159	118/210	38/73	1.19 (0.83–1.70); *P*: 0.354	1.14 (0.70–1.85); *P*: 0.607	1.17 (0.84–1.65); *P*: 0.359	1.03 (0.67–1.59); *P*: 0.900
Age							
< 60	81/163	114/226	43/86	1.01 (0.70–1.46); *P*: 0.948	1.05 (0.66–1.69); *P*: 0.836	1.02 (0.72–1.44); *P*: 0.911	1.04 (0.68–1.59); *P*: 0.847
≥ 60	91/207	153/255	39/92	1.45 (1.04–2.02); P: 0.030	1.09 (0.68–1.74); *P*: 0.729	1.35 (0.99–1.86); *P*: 0.061	0.87 (0.57–1.33); *P*: 0.530
Smoking status							
Never	106/295	165/380	46/152	1.24 (0.92–1.66); *P*: 0.155	0.91 (0.61–1.37); *P*: 0.654	1.15 (0.87–1.52); *P*: 0.340	0.80 (0.56–1.16); *P*: 0.242
Ever	66/75	102/101	36/26	1.19 (0.77–1.83); *P*: 0.440	1.61 (0.88–2.97); *P*: 0.125	1.27 (0.84–1.93); *P*: 0.253	1.46 (0.84–2.53); *P*: 0.183
Alcohol consumption							
Never	149/331	225/449	70/168	1.12 (0.87–1.46); *P*: 0.387	0.99 (0.69–1.41); *P*: 0.944	1.08 (0.85–1.39); *P*: 0.520	0.92 (0.67–1.27); *P*: 0.615
Ever	23/39	42/32	12/10	2.27 (1.11–4.60); P: 0.024	2.23 (0.81–6.13); *P*: 0.121	2.26 (1.15–4.43); P: 0.018	1.40 (0.56–3.54); *P*: 0.472
BMI (kg/m^2^)							
< 24	112/212	173/250	52/84	1.29 (0.9–1.76); *P*: 0.113	1.18 (0.77–1.81); *P*: 0.459	1.26 (0.94–1.69); *P*: 0.131	1.02 (0.69–1.51); *P*: 0.932
≥ 24	60/158	94/231	30/94	1.11 (0.75–1.65); *P*: 0.610	0.95 (0.56–1.60); *P*: 0.847	1.07 (0.73–1.55); *P*: 0.744	0.89 (0.56–1.42); *P*: 0.631

^a^ The genotyping was successful in 521 (100.00%) NSCLC cases, and 1029 (99.90%) controls for *CTLA-4* rs733618 T>C; ^b^ Adjusted for age, sex, BMI, smoking status and alcohol consumption (besides stratified factors accordingly) in a logistic regression model.

### SNP haplotypes

Using SHESIS online haplotype construction software [( http://analysis.bio-x.cn/myAnalysis.php), Bio-X Inc., Shanghai, China] [13], we constructed four haplotypes (Table 9). We found there was no difference in haplotype distribution among NSCLC cases and the control subjects (Table 9).

**Table 9:** *CTLA4* haplotype frequencies (%) in cases and controls and risk of NSCLC

Haplotypes	case (*n* = 1042)		control (*n* = 2060)		Crude OR (95% CI)	*P*
	*n*	%	*n* (%)	%		
C G G C	430	41.35	836	40.66	Reference	
C G G T	285	27.40	595	28.94	0.93 (0.78–1.12)	0.445
C A A T	189	18.17	374	18.19	0.98 (0.80–1.21)	0.869
T A G T	123	11.83	239	11.62	1.00 (0.78–1.28)	0.996
Others	13	1.25	12	0.58	2.11 (0.95–4.66)	0.060

With the order of *CTLA4* rs16840252 C>T, rs231775 G>A, rs3087243 G>A and rs733618 T>C in gene position.

### The power of the present study (α= 0.05)

For *CTLA-4* rs733618 T>C, the power value was 0.629 in additive model among ≥ 60 years subgroup, and 0.651 in additive model and 0.692 in dominant model among even drinking subgroup.

## DISCUSSION

In generally, immune escape may be an important mechanism in the development of malignancies. The costimulatory signal related SNPs, common variants among individuals, may play important roles in the development of human cancers. In the present study, we explored the effect of *CTLA-4* tagging SNPs in NSCLC among Eastern Chinese Han population for the first time. We found that *CTLA-4* tagging polymorphisms might be not correlated with the susceptibility of overall NSCLC. The results of haplotype analysis suggested that there was no difference in haplotype distribution among NSCLC cases and the control subjects. However, in the stratified analyses by age, sex, BMI, alcohol use and smoking status, we found that *CTLA-4* rs733618 T>C polymorphism was associated with the development of NSCLC in ≥ 60 years and even drinking subgroups.

NSCLC is a multifactorial disease which results from the interaction between individual's genetic backgrounds and environmental risk factors. Previous studies have demonstrated that *CTLA-4* rs733618 T>C polymorphism decreases a transcription factor binding site for nuclear factor 1 and weaken CTLA-4 expression on cell surface [14, 15]. Accumulating evidences suggested that *CTLA-4* rs733618 T>C polymorphism might be associated with the increased risk of systemic lupus erythematosus [16–19]. However, the relationship of *CTLA-4* rs733618 T>C polymorphism with the development of cancer was conflicting. With an interest in the correlation of *CTLA-4* rs733618 T>C polymorphism with cancer susceptibility, a case-control study explored the hypothesis that *CTLA-4* rs733618 T>C polymorphism was associated with the etiology of NSCLC [10]; however, this study in an Iranian population established null association between *CTLA-4* rs733618 T>C polymorphism and NSCLC. In the current study, we found that *CTLA-4* rs733618 T>C polymorphism was associated with the development of NSCLC. In addition, a previous study in China conducted by Li *et al.*, compared with the *CTLA-4* rs733618 T allele, the C allele increased the risk of breast cancer [20]. Our findings might be supported by this study. To the best of our knowledge, the present case-control study was the first study to examine the potential relationship between *CTLA-4* rs733618 T>C polymorphism and the development of NSCLC in Asians. However, due to the limited sample size, these potential association should be explained with very caution. In the future, more case-control studies with detailed lifestyle and environmental factors data should be conducted to explore these potential association.

We should acknowledge some limitations in this case-control study. Firstly, the NSCLC patients and control subjects were enrolled from local hospitals in Eastern China and might not fully represent the general Chinese Han population. Secondly, the moderate sample size of NSCLC cases might limit the statistical power to obtain a real assessment, especially in the stratified analyses. Further well-designed fine-mapping studies with large sample sizes are needed to confirm our findings. Thirdly, the clinical information on metastasis and survival of NSCLC could not be derived till now, which restricted further explores on the potential role of *CTLA-4*tagging polymorphisms in NSCLC progression and prognosis. Fourthly, the information about family cancer history was not collected. Finally, due to lack of the information about individual's lifestyle, further determination for the interactions of gene-gene and gene-environment were not carried out. In consideration of the complex pathological process of NSCLC, these gene and environmental risk factors should not be ignored.

In summary, the present case-control study highlights that *CTLA-4* rs733618 T>C polymorphism was associated with the development of NSCLC in ≥ 60 years and even drinking subgroups. In addition, a larger population-based fine-mapping study as well as detailed functional assessment are necessary to confirm or refute our findings.

## MATERIALS AND METHODS

### Study population and patient selection

A total of 521 Eastern Chinese Han population with NSCLC were included in this study. These NSCLC patients were diagnosed from January 2014 to December 2016 at the Affiliated People's Hospital of Jiangsu University (Zhenjiang, China) and the Affiliated Union Hospital of Fujian Medical University (Fuzhou, China). Diagnosis of NSCLC was confirmed via histopathological examinations after operation or bronchoscope check. The selection criteria of NSCLC cases were: (1) sporadic NSCLC cases; (2) NSCLC patients without any treatment and (3) Eastern Chinese Han population. And the corresponding major exclusion criteria were: (1) autoimmune disease history; (2) NSCLC cases who received prior chemoradiotherapy and targeted threapy and (3) a history of another malignancy. Meanwhile, a total of 1,030 non-cancer controls were enrolled when they attended a routine physical examination in the Physical Examination Center of these hospitals. Additionally, the criteria for non-cancer controls selection was: (1) cancer-free subjects; (2) without autoimmune disease; (3) sex and gender matched to NSCLC patients; (4) unrelated subjects and (5) Eastern Chinese Han population. During recruitment, all study subjects signed the written informed consents following the Declaration of Helsinki. The information of risk factors and demographics was obtained by a pre-structured questionnaire. NSCLC cases and controls were well-matched in terms of age and sex (Table 1). Subjects who smoked at least one cigarette per day over 1 year were considered as ‘ever smokers’ [21], and those who drinked no less than three times a week for more than 6 months were defined as ‘ever drinkers’ [21]. The Ethical Committee of Fujian Medical University approved the study protocols (No. 2017KY019).

### Selection of CTLA-4 tagging SNPs

The *CTLA-4* tagging SNPs were selected through the Genome Variation Server (GVS) data (http://gvs.gs.washington.edu/GVS147/). The major included criterion were: (a) *P* value of HWE ≥ 0.05, (b) minor allele frequency (MAF) ≥ 0.05 (c) pairwise linkage disequilibrium (LD) *r*^2^ threshold of 0.8 between SNPs (*r*^2^ > 0.8) and (d) the call rate ≥ 95 % in the CHB cohort were included [22]. Finally, *CTLA-4* rs3087243 G>A, rs16840252 C>T, rs733618 T>C and rs231775 G>A polymorphisms were eligible for study. Table 2 summarizes the information of the selected SNPs.

### DNA extraction and genotyping

Two milliliters blood sample was donated by each enrolled subject and stored in Ethylenediamine tetraacetic acid (EDTA)-anticoagulation tube. DNA was elaborately extracted from lymphocytes by using the Promega kit (Promega, Madison, USA). We extracted DNA according to the manufacturer's instruction ( www.promega.com/protocols/). The genotypes of *CTLA-4* rs16840252 C>T, rs231775 G>A, rs3087243 G>A and rs733618 T>C polymorphisms were evaluated using a custom-by-design 48-Plex SNPscan Kit (Genesky Biotechnologies Inc., Shanghai, China) as presented in previous studies [23, 24]. Briefly, we first denatured 150ng DNA sample at 98°C for 5 min. The ligation reaction was performed in an ABI 2720 thermal cycler. A 48-plex fluorescence PCR reaction was carried out for each ligation product. Then, in an ABI 3730XL sequencer, the obtained PCR products were separated and detected by using capillary electrophoresis. The raw data were analyzed by GeneMapper 4.1 software (Applied Biosystems, USA). A 4% randomly selected DNA sample was reciprocally verified by another laboratory technician, and the reproducibility was 100%.

### Statistical analysis

Statistical analysis of this case-control study was done using the SAS 9.4 Statistical Package for Windows (SAS Institute, Cary, NC). A *P* < 0.05 (two–tailed) was accepted as the level of significance. The results of continuous variables were presented as mean ± standard deviation (SD). The mean values of age between NSCLC patients and non-cancer controls were calculated using the Student's *t*-test. The deviation of HWE in controls was analyzed using an online Pearson's two-sided χ^2^ test (http://ihg.gsf.de/cgi-bin/hw/hwa1.pl) [25]. The differences in smoking, drinking, demographic variables and the frequencies of genotypes between NSCLC cases and controls were also determined by χ^2^ test. The crude/adjusted ORs and the corresponding 95% CIs were harnessed to assess the relationship of *CTLA-4* rs16840252 C>T, rs231775 G>A, rs3087243 G>A and rs733618 T>C polymorphism genotypes with NSCLC risk. SHESIS online haplotype construction software [(http://analysis.bio-x.cn/myAnalysis.php), Bio-X Inc., Shanghai, China] was used to obtain the haplotypes [13]. The power value of this study was calculated by Power and Sample Size software (http://biostat.mc.vanderbilt.edu/twiki/bin/view/Main/PowerSampleSize) [26].
